# Stress-related eating, obesity and associated behavioural traits in adolescents: a prospective population-based cohort study

**DOI:** 10.1186/1471-2458-14-321

**Published:** 2014-04-07

**Authors:** Anne Jääskeläinen, Nina Nevanperä, Jouko Remes, Fanni Rahkonen, Marjo-Riitta Järvelin, Jaana Laitinen

**Affiliations:** 1 Finnish Institute of Occupational Health, P.O. Box 310, FI-70101 Kuopio, Finland; 2 Finnish Institute of Occupational Health, Oulu, Finland; 3 Biocenter Oulu, University of Oulu, Oulu, Finland; 4 Institute of Health Sciences, University of Oulu, Oulu, Finland; 5 National Institute for Health and Welfare, Oulu, Finland; 6 Department of Epidemiology and Biostatistics, MRC Health Protection Agency Centre for Environment and Health, School of Public Health, Imperial College London, London, UK; 7 Unit of Primary Care, Oulu University Hospital, Oulu, Finland

**Keywords:** Adolescent, Body mass index, Cohort studies, Diet, Drinking behaviour, Health behaviour, Latent class analysis, Obesity, Psychological stress

## Abstract

**Background:**

Stress-related eating is associated with unhealthy eating and drinking habits and an increased risk of obesity among adults, but less is known about factors related to stress-driven eating behaviour among children and adolescents. We studied the prevalence of stress-related eating and its association with overweight, obesity, abdominal obesity, dietary and other health behaviours at the age of 16. Furthermore, we examined whether stress-related eating is predicted by early-life factors including birth size and maternal gestational health.

**Methods:**

The study population comprised 3598 girls and 3347 boys from the Northern Finland Birth Cohort 1986 (NFBC1986). Followed up since their antenatal period, adolescents underwent a clinical examination, and their stress-related eating behaviour, dietary habits and other health behaviours were assessed using a postal questionnaire. We examined associations using cross-tabulations followed by latent class analysis and logistic regression to profile the adolescents and explain the risk of obesity with behavioural traits.

**Results:**

Stress-related eating behaviour was more common among girls (43%) than among boys (15%). Compared with non-stress-driven eaters, stress-driven eaters had a higher prevalence of overweight, obesity and abdominal obesity. We found no significant associations between stress-eating and early-life factors. Among girls, tobacco use, shorter sleep, infrequent family meals and frequent consumption of chocolate, sweets, light sodas and alcohol were more prevalent among stress-driven eaters. Among boys, the proportions of those with frequent consumption of sausages, chocolate, sweets, hamburgers and pizza were greater among stress-driven eaters. For both genders, the proportions of those bingeing and using heavy exercise and strict diet for weight control were higher among stress-eaters. Besides a ‘healthy lifestyle’ cluster, latent class analysis revealed two other patterns (‘adverse habits’, ‘unbalanced weight control’) that significantly explained the risk of overweight among boys and girls.

**Conclusions:**

Stress-related eating is highly prevalent among 16-year-old girls and is associated with obesity as well as adverse dietary and other health behaviours among both genders, but intrauterine conditions are seemingly uninvolved. In terms of obesity prevention and future health, adolescents who use eating as a passive way of coping could benefit from learning healthier strategies for stress and weight management.

## Background

Obesity among children and adolescents has reached epidemic proportions and stress-induced food and drink intake, i.e. eating or drinking in response to a stressful event in order to feel better, may be one factor contributing to the phenomenon [1]. Stress-related eating has been linked to an increased risk of obesity and distinct features in food and alcohol consumption among adults [2,3], but only a few studies exist on the relationship of stress with body weight or eating behaviour among paediatric and adolescent populations. Recently, De Vriendt et al. reported an association between perceived stress and both general and abdominal obesity among female adolescents [4]. Previously, Michaud and colleagues observed that among high-school students, dietary fat and total energy intake were higher on the day of a school examination [5]. Adverse eating habits, such as intake of high energy-dense (fatty) foods, snacking, skipping breakfast and eating less fruit and vegetables, seem to be more common among children and adolescents experiencing stress [6,7] and those prone to emotional eating, i.e., overeating in response to negative emotional arousal [8].

Very little data exist on lifestyle factors and health behaviours in relation to stress-induced eating and drinking in youth. In general, weight-related problems seem to cluster among individuals: overweight, binge eating (i.e. eating a large amount of food in a short period of time associated with a sense of loss of control over eating during the episode) and extreme weight-control behaviours often co-occur among both boys and girls [9]. Among obese individuals, binge eating is frequently accompanied by stress-management deficit [10]. Preliminary evidence suggests that eating with one’s family could have a protective role in disordered eating behaviour among adolescent girls [11,12].

As for the origins of stress vulnerability and ways of responding to stress, intrauterine conditions could be influential. The offspring of mothers who are stressed while pregnant are more likely to have cognitive and emotional problems [13-15], and premature birth and small birth size are shown to reflect foetal exposure to elevated levels of maternal gestational stress [16-18]. In addition, prenatal alcohol exposure is found to predispose to cognitive, neuropsychological and behavioural problems later in life [19]. The role of other potential stressors or stress indicators related to maternal gestational health behaviour and well-being, such as smoking and either insufficient or excessive weight gain *per se*, in offspring’s neurobehavioural development has not been established [20-22].

In the present study, we investigated the prevalence of self-reported stress-related eating behaviour and its association with overweight/obesity, abdominal obesity, food and alcohol consumption and other health behaviours (tobacco use, sleeping, physical activity, eating meals with one’s family, binge eating and extreme weight control practices) among 16-year-old boys and girls. Furthermore, we examined whether exposure to stress *in utero* increases vulnerability to stress-related eating at the age of 16. Stress indicators included the following early-life factors: maternal gestational weight gain, smoking and alcohol use in early pregnancy, and the subject’s own birth size.

## Methods

### Study population

The study population originated from the population-based Northern Finland Birth Cohort 1986 (NFBC1986), which comprises 9432 live born children whose due dates were between 1 July 1985 and 30 June 1986 [23]. The NFBC1986 covered 99% of births in the two northernmost provinces of Finland, Oulu and Lapland. The mothers and their offspring have been studied prospectively since the 12th gestational week and the most recent follow-up was carried out in 2001–2002 at offspring age 16 years. Of those adolescents alive and traceable at the 16-year follow-up (n = 9215), 7344 (80%) responded to a postal questionnaire inquiring about their health and well-being, and 6798 (74%) participated in a clinical examination including anthropometric measurements. Here, we analysed 6945 adolescents (3598 girls, 3347 boys) for whom data on stress-related eating behaviour were available.

### Ethics statement

The Ethics Committees of the Northern Ostrobothnia Hospital District (1985) and the Faculty of Medicine of the University of Oulu (2000) approved the study and we obtained written informed consent from each participant according to the Declaration of Helsinki.

### Data collection and study variables

#### Body mass index-based and waist circumference-based obesity

Trained nurses carried out clinical examinations according to a standardised protocol in municipalities of Northern Finland and also in major cities elsewhere in Finland. Height measurement was recorded on a study form to one decimal place and weight was measured using a calibrated scale to the nearest 0.1 kg with subjects in their underwear. Waist circumference was measured at the level midway between the lowest rib margin and the iliac crest. Body mass index (BMI) was calculated as weight/height^2^ (kg/m^2^) and adolescents were classified as normal weight/underweight, overweight and obese according to the International Obesity Task Force age- and gender-specific BMI cut-off values [24]. The BMI cut-off points for overweight and obesity (corresponding to adult BMI of 25.0 and 30.0 kg/m^2^) were 23.90 and 28.88 kg/m^2^ for 16-year-old boys and 24.37 and 29.43 kg/m^2^ for 16-year-old girls. Abdominal obesity was defined using the International Diabetes Federation (IDF) paediatric criteria for metabolic syndrome, i.e., waist circumference ≥90th percentile or adult cut-off if lower [25]. In the NFBC1986 data the waist circumference 90th percentile among girls was 82.0 cm; therefore we used the IDF adult cut-off (80 cm for European women) to define abdominal obesity among females.

#### Stress-related eating

Stress-related eating behaviour at 16 years was based on an item in the revised Ways of Coping Checklist (WCC) [26,27]. WCC is a questionnaire containing a wide range of coping thoughts and actions that people may engage in when under stress, and it is used as a measure to study coping processes. Stress-related eating was assessed using the item “When you encounter stress in life, e.g. a difficult matter, occasion or situation, do you try to make yourself feel better by eating?”. Those who answered “to some extent”, “fairly often” or “very often” were classified as stress-driven eaters. Those who answered “never” were classified as non-stress-driven eaters.

#### Indicators of early-life stress

Data on maternal health behaviours and well-being before conception and in early pregnancy (alcohol use, smoking habits, weight) were collected via questionnaires given to all mothers at their first antenatal visit to the maternal welfare clinic, i.e., at 12 weeks of gestation at the latest, and they returned them by the 24th week of gestation. Maternal alcohol use and smoking were assessed using the following questions: ‘Have you drank alcohol during this pregnancy (yes/no)?’ and ‘The number of cigarettes after the 2nd month of pregnancy (none/1–9/>9)?’. Gestational weight gain was calculated as the difference between weight at the 20th week of pregnancy and self-reported pre-pregnancy weight and was classified into fourths based on the following quartile cut-off values: Q1 ≤ 3.0 kg; Q2 > 3.0 kg and ≤5.0 kg; Q3 > 5.0 kg and ≤7.0 kg; Q4 > 7.0 kg, as previously described [28]. Using data on gestational age and weight at birth recorded by the attending midwives at the delivery hospital, offspring size at birth was classified as appropriate, small and large for gestational age, defined respectively as birth weight between the 10th and 90th, below the 10th, and over the 90th percentile of the gender- and gestational age-specific cohort distributions. Pre-term birth was defined as childbirth before 37 weeks of gestation and birth weight of less than 2500 grams and 4000 grams or more were considered low and high birth weight, respectively.

#### Food and drink consumption at 16 years

Consumption frequencies of food and non-alcoholic and alcoholic beverages were elicited using a postal questionnaire sent to all traced adolescents at the 16-year follow-up. Adolescents were asked to consider their food and non-alcoholic beverage consumption during the previous six months and to select a response alternative on a six-point scale ranging from ‘never or less often than once a month’ to ‘once a day or more often”. The frequency of alcohol consumption was measured on a six-point scale ranging from ‘I have never drunk’ to ‘I use alcohol once a week or more often’. Based on previous findings on stress-related food and drink preferences among adults and adolescents, we chose snack-type fatty, salty and sweet foods and sugary, sugar-free and alcoholic beverages for the analyses [2,6,8,29,30]. Frequent intake of sausages/frankfurters was defined as twice a week or more often, for other foods and non-alcoholic beverages frequent consumption was defined as 3–5 times a week or more often. Alcohol intake was classified as ‘never’, ‘randomly’ and ‘once a month or more often’.

#### Other health behaviours at 16 years

We collected information on adolescents’ other health behaviours including tobacco use, physical activity, sleeping habits, binge eating, weight control practices and eating meals with one’s family via a postal questionnaire at 16-year follow-up. The exact wordings of the questions were: “Have you ever smoked or used snuff in your life?”; “Have you ever smoked or used snuff regularly (i.e. at least one cigarette or a pinch of snuff almost daily for a year or longer)?”; “Do you currently smoke?”; “Do you currently use snuff?”; “Outside the school hours and the time spent on school trips, how many hours a week altogether do you spend in strenuous physical activity (getting out of breath and sweating at least slightly)?”; “How many hours do you usually sleep per day (24 hours)?”; “How often do you devour large amounts of food?”; “Do you use heavy exercise to control your weight?”; “Do you use strict diet or fasting to control your weight?”; “How often do you have your meals with your parents or other family members?”. Tobacco use was classified as ‘never smoked or taken snuff’, ‘experimental or occasional use of cigarettes or snuff’ and ‘regular use of cigarettes or snuff’. Weekly amount of physical activity was classified into less than 2 hours, 2–3 hours and more than 3 hours of moderate- to vigorous-intensity exercise. Categories for sleep duration were less than 8 hours, 8–9 hours and more than 9 hours of sleep per night. Binge eating comprised seven response categories (from ‘never’ to ‘daily’) and was defined as devouring large amounts of food at least once a month. The questions on weight control practices, i.e. heavy exercise or strict diet/fasting, consisted of three response categories: ‘never’, ‘occasionally’ and ‘often’, which were combined into ‘never’ and ‘occasionally or often’ for the analyses. Eating with one’s family infrequently was defined as having meals with parents or other family members a few times a week or less often, in contrast to having family meals frequently i.e. daily or almost daily.

#### Family characteristics

We used self-reported maternal basic education level, i.e. comprehensive school or upper secondary school, as an indicator of family’s socio-economic status. In addition, parents’ attentiveness towards their child was measured with an item included in the questionnaire for adolescents: ‘Are your parents interested in your schooling, hobbies or other things that are important to you (never/seldom/almost always)?’

### Statistical analyses

All analyses were gender-stratified. The statistical significance of the differences in the prevalence of obesity, early-life factors, eating and drinking habits and health behaviours between stress-driven and non-stress-driven eaters was estimated using Pearson’s chi-squared test. To compare differences in waist circumference and BMI between the groups, we used 95% confidence intervals (95% CI) for the means. Furthermore, we employed latent class analysis (LCA) which is a statistical modelling technique for identifying unobservable subgroups within populations by assessing observed response patterns. LCA was used firstly to assign the individuals to the most likely latent class(es) based on their behavioural characteristics. We then evaluated the goodness of fit with the observed data for a set of five candidate models, i.e., models with one to five classes. To determine the best-fitting solution, we used the Akaike information criterion (AIC), Bayesian information criterion (BIC), and sample-size adjusted BIC (SSABIC) as the measures of goodness-of-fit. Lower values of these fit indices indicate a better fit [31]. In addition, the Lo-Mendell-Rubin likelihood ratio test (LMR) and entropy measure were used for identifying the optimal latent class solution; a low LMR *p*-value (< 0.05) indicates that the model fits the measured data better than its more parsimonious (reduced) alternative. Entropy depicts the average accuracy of assigning individuals into latent classes based on the posterior class membership probabilities. Entropy values range from 0 to 1 with higher values indicating greater precision of classification. Finally, associations of identified latent classes with obesity measures were assessed using multinomial logistic regression analysis adjusted for maternal basic education level and parental interest in their child’s hobbies and schooling.

The results are reported as frequencies and percentages, and, for continuous variables, as means and 95% CI. In addition, we present both crude and adjusted odds ratios and their 95% CI from logistic regression analysis. We conducted LCA using Mplus Version 6.11 software, and performed the other statistical analyses using IBM SPSS Statistics for Windows, Version 19.0 (IBM Corp., Armonk, NY, USA). Due to differences in response rates between modes of data collection, the number of subjects in the tables varies.

## Results

Stress-driven eating was more prevalent among girls (43%) than among boys (15%) (Table 1). However, the majority of stress-eaters fell under the category of ‘to some extent’ (39% of girls and 14% of boys) and only 4% of girls and 1% of boys reported that they used the passive way of coping either ‘fairly often’ or ‘very often’. Abdominal obesity was more common among girls (13%) than among boys (10%), whereas 14% of girls and 17% of boys were either overweight or obese.

**Table 1:** Characteristics of the Northern Finland Birth Cohort 1986 study population (16-year follow-up data collected in 2001–2002)

	**Girls n = 3598**		**Boys n = 3347**		***p *** **(χ**^**2**^**-test)**
	**%**	**Mean (95% CI)**	**%**	**Mean (95% CI)**	
**Weight (kg)**		56.9 (56.6, 57.3)		64.6 (64.2, 65.1)	
**Height (cm)**		163.9 (163.7, 164.1)		174.6 (174.4, 174.9)	
**Body mass index (kg/m**^**2**^**)**		21.2 (21.1, 21.3)		21.1 (21.0, 21.3)	
**Waist circumference (cm)**		71.9 (71.6, 72.2)		75.8 (75.4, 76.1)	
**Overweight**	10.9		12.7		
**Obesity**	2.9		4.3		<0.001
**Abdominal obesity**	13.2		10.4		0.001
**Eating in a stressful situation to make oneself feel better**					
Never	56.8		84.8		
To some extent	39.2		14.3		
Fairly often	3.2		0.8		
Very often	0.8		0.2		<0.001
**Physical activity hours per week**					
<2	40.7		31.3		
2–3	29.9		23.3		
>3	29.5		45.3		<0.001
**Use of tobacco**					
Never	33.2		36.9		
Experimental or occasional	39.9		38.9		
Regular	26.9		24.3		0.002
**Sleeping hours per night**					
<8	24.4		16.7		
8–9	65.2		66.3		
>9	10.4		16.9		<0.001
**Devouring large amounts of food**					
Never	46.3		42.4		
Hardly ever	23.1		20.9		
Occasionally	21.6		26.3		
Once a month	3.5		3.5		
Once a week	3.3		3.4		
2–3 times a week	1.7		2.0		
Daily	0.6		1.5		<0.001
**Strict diet to control weight**					
Never	70.0		90.1		
Occasionally	27.7		9.4		
Often	2.3		0.5		<0.001
**Heavy exercise to control weight**					
Never	57.4		69.8		
Occasionally	37.5		22.5		
Often	5.1		7.6		<0.001
**Meals with parents or other family members**					
Daily or almost daily	63.5		75.0		
A few times a week	17.3		14.0		
1–2 times a week	7.6		5.2		
Occasionally	8.8		4.6		
Hardly ever	2.7		1.2		<0.001
**Maternal basic education**					
Comprehensive school	67.0		68.2		
Upper secondary school	33.0		31.8		0.309
**Parents interested in their child’s schooling and hobbies**					
Never	0.8		0.5		
Seldom	13.8		14.0		
Almost always	85.4		85.5		0.372

Stress-driven eaters had greater BMI and waist circumferences as well as a higher prevalence of overweight, obesity and abdominal obesity than non-stress-driven eaters (Table 2). The prevalence of overweight and obesity combined was 15% among stress-eater girls and 12.5% among non-stress-eater girls, and 21.5% among stress-eater boys and 16% among non-stress-eater boys.

**Table 2:** Association between stress-driven eating behaviour and obesity measures (body mass index, waist circumference, overweight, obesity and abdominal obesity)

**Measure or degree of obesity**	**Girls**					**Boys**				
	**Non-stress-driven eaters n = 2026**		**Stress-driven eaters n = 1538**			**Non-stress-driven eaters n = 2757**		**Stress-driven eaters n = 496**		
	**Mean (95% CI)**	**n (%)**	**Mean (95% CI)**	**n (%)**	***p *** **(χ**^**2**^**-test)**	**Mean (95% CI)**	**n (%)**	**Mean (95% CI)**	**n (%)**	***p *** **(χ**^**2**^**-test)**
**Body mass index (kg/m**^**2**^**)**	21.0 (20.8, 21.1)		21.4 (21.3, 21.6)			21.0 (20.9, 21.1)		21.8 (21.4, 22.1)		
**Waist circumference (cm)**	71.4 (71.1, 71.8)		72.6 72.1, 73.0)			75.5 (75.1, 75.8)		77.2 (76.2, 78.2)		
**Normal weight + underweight**		1757 (87.4)		1296 (84.7)			2285 (83.8)		388 (78.5)	
**Overweight**		208 (10.3)		177 (11.6)			336 (12.3)		77 (15.6)	
**Obesity**		45 (2.2)		58 (3.8)	*0.010*		106 (3.9)		29 (5.9)	*0.012*
**Abdominal obesity**		215 (12.1)		198 (14.7)			236 (9.7)		58 (13.5)	
**No abdominal obesity**		1566 (87.9)		1152 (85.3)	*0.037*		2194 (90.3)		371 (86.5)	*0.020*

As shown in Table 3, stress-related eating behaviour was not associated with any prenatal (maternal gestational weight gain, alcohol use and smoking) or perinatal factor (pre-term birth, birth weight and gestational age-adjusted birth weight).

**Table 3:** Association between stress-driven eating behaviour and early-life factors

**Early-life factor**		**Girls**			**Boys**		
		**Non-stress-driven eaters n (%)**	**Stress-driven eaters n (%)**	***p *** **(χ**^**2**^**-test)**	**Non-stress-driven eaters n (%)**	**Stress-driven eaters n (%)**	***p *** **(χ**^**2**^**-test)**
**Maternal smoking at the end of the 2nd month of pregnancy**	No	1602 (81.2)	1226 (81.2)		2176 (80.7)	412 (84.4)	
	1-9 cigarettes a day	173 (8.8)	139 (9.2)		255 (9.5)	36 (7.4)	
	>9 cigarettes a day	197 (10.0)	144 (9.5)	*0.836*	267 (9.9)	40 (8.2)	*0.142*
**Maternal alcohol use during pregnancy**	No	1738 (87.4)	1299 (85.9)		2346 (87.0)	432 (88.3)	
	Yes	251 (12.6)	214 (14.1)	*0.192*	352 (13.0)	57 (11.7)	*0.420*
**Maternal weight gain during the first 20 weeks of pregnancy**	Lowest quartile	551 (29.7)	391 (27.9)		653 (25.9)	113 (24.8)	
	Second quartile	556 (30.0)	455 (32.5)		726 (28.8)	148 (32.5)	
	Third quartile	442 (23.8)	330 (23.6)		628 (24.9)	116 (25.4)	
	Highest quartile	306 (16.5)	224 (16.0)	*0.449*	511 (20.3)	79 (17.3)	*0.298*
**Birth weight**	Low <2500 g	81 (4.0)	43 (2.8)		76 (2.8)	14 (2.8)	
	Normal 2500–3999 g	1613 (79.6)	1238 (80.5)		2054 (74.5)	345 (69.6)	
	High ≥4000 g	332 (16.4)	257 (16.7)	*0.152*	627 (22.7)	137 (27.6)	*0.059*
**Gestational age at birth**	<37 weeks (preterm)	91 (4.5)	61 (4.0)		134 (4.9)	21 (4.2)	
	≥37 weeks	1935 (95.5)	1477 (96.0)	*0.453*	2621 (95.1)	475 (95.8)	*0.647*
**Birth weight for gestational age**	Small	185 (9.1)	127 (8.3)		237 (8.6)	44 (8.9)	
	Appropriate	1640 (80.9)	1250 (81.3)		2262 (82.0)	396 (79.8)	
	Large	201 (9.9)	161 (10.5)	*0.597*	258 (9.4)	56 (11.3)	*0.384*

As regards the consumption of snack-type foods and beverages (Table 4), among boys, the proportions of those who ate sausages, hamburgers and pizzas, chocolate and sweets several times per week were higher among stress-driven eaters than among non-stress-driven eaters. Among girls, frequent consumption of chocolate, sweets, and sugar-free soft drinks was more common among stress-eaters than non-stress-eaters. As for differences in alcohol consumption between the two groups, a higher proportion of stress-eaters drank alcohol frequently, i.e., on a monthly basis. No differences in sugar-sweetened drink consumption were seen.

**Table 4:** Association between stress-driven eating behaviour, consumption of sweet or salty snack-type foods and beverages and other health behaviours

**Food or beverage**	**Amount or frequency**	**Girls**			**Boys**		
		**Non-stress-driven eaters n (%)**	**Stress-driven eaters n (%)**	***p *** **(χ**^**2**^**-test)**	**Non-stress-driven eaters n (%)**	**Stress-driven eaters n (%)**	***p *** **(χ**^**2**^**-test)**
**Sausages and frankfurters**	Once a week or less often	1692 (84.2)	1278 (83.6)		1890 (69.3)	313 (63.7)	
	Twice a week or more often	317 (15.8)	251 (16.4)	*0.611*	838 (30.7)	178 (36.3)	*0.018*
**Hamburgers and pizzas**	Twice a week or less often	1959 (97.5)	1480 (96.9)		2519 (92.4)	438 (89.0)	
	3–5 times a week or more often	50 (2.5)	48 (3.1)	*0.256*	208 (7.6)	54 (11.0)	*0.015*
**Chocolate**	Twice a week or less often	1788 (89.0)	1303 (85.2)		2482 (91.2)	433 (88.0)	
	3–5 times a week or more often	221 (11.0)	227 (14.8)	*0.001*	239 (8.8)	59 (12.0)	*0.028*
**Sweets**	Twice a week or less often	1481 (74.0)	1019 (67.0)		2105 (77.6)	357 (72.7)	
	3-5 times a week or more often	520 (26.0)	503 (33.0)	*<0.001*	607 (22.4)	134 (27.3)	*0.020*
**Ice cream**	Twice a week or less often	1509 (75.0)	1115 (73.0)		2054 (75.2)	363 (74.2)	
	3–5 times a week or more often	503 (25.0)	413 (27.0)	*0.175*	676 (24.8)	126 (25.8)	*0.650*
**Sugar-free soft drinks (light sodas)**	Twice a week or less often	1805 (90.2)	1336 (88.0)		2456 (90.5)	435 (88.6)	
	3–5 times a week or more often	197 (9.8)	182 (12.0)	*0.042*	259 (9.5)	56 (11.4)	*0.216*
**Sugar-sweetened soft drinks**	Twice a week or less often	1552 (77.2)	1173 (76.8)		1799 (66.0)	302 (61.5)	
	3–5 times a week or more often	459 (22.8)	354 (23.2)	*0.809*	925 (34.0)	189 (38.5)	*0.057*
**Alcoholic beverages**	Never	1047 (51.9)	615 (40.2)		1565 (57.1)	267 (54.0)	
	Randomly	544 (26.9)	439 (28.7)		666 (24.3)	115 (23.3)	
	Once a month or more often	428 (21.2)	475 (31.1)	*<0.001*	511 (18.6)	112 (22.7)	*0.111*
**Physical activity**	<2 hours a week	796 (39.7)	637 (41.9)		852 (31.3)	152 (31.0)	
	2–3 hours a week	613 (30.6)	440 (28.9)		627 (23.0)	116 (23.6)	
	>3 hours a week	596 (29.7)	444 (29.2)	*0.391*	1246 (45.7)	223 (45.4)	*0.956*
**Tobacco use**	Never smoked or taken snuff	758 (37.5)	417 (27.2)		1015 (37.2)	166 (33.6)	
	Experimental or occasional use of cigarettes or snuff	763 (37.8)	657 (42.9)		1058 (38.7)	198 (40.1)	
	Smoking or taking snuff regularly	499 (24.7)	458 (29.9)	*<0.001*	658 (24.1)	130 (26.3)	*0.288*
**Sleeping**	<8 hours per night	450 (22.4)	410 (26.8)		446 (16.4)	92 (18.9)	
	8–9 hours per night	1344 (66.9)	966 (63.1)		1813 (66.8)	312 (63.9)	
	>9 hours per night	215 (10.7)	154 (10.1)	*0.010*	457 (16.8)	84 (17.2)	*0.370*
**Binge eating**	Never - occasionally	1914 (94.8)	1316 (85.8)		2513 (91.3)	395 (79.6)	
	Once a month - daily	104 (5.2)	218 (14.2)	*<0.001*	239 (8.7)	101 (20.4)	*<0.001*
**Strict diet to control weight**	Never	1544 (76.7)	928 (61.0)		2487 (91.3)	399 (83.1)	
	Occasionally or often	469 (23.3)	594 (39.0)	*<0.001*	237 (8.7)	81 (16.9)	*<0.001*
**Heavy exercise to control weight**	Never	1284 (64.0)	738 (48.6)		1963 (71.8)	286 (58.5)	
	Occasionally or often	723 (36.0)	781 (51.4)	*<0.001*	770 (28.2)	203 (41.5)	*<0.001*
**Eating meals with family**	Daily or almost daily	1342 (66.6)	918 (59.8)		2059 (75.4)	361 (73.2)	
	A few times a week or less often	674 (33.4)	616 (40.2)	*<0.001*	672 (24.6)	132 (26.8)	*0.309*

Stress-driven eating was associated with a broader range of health behaviours among girls than among boys (Table 4). Among both boys and girls with stress-related eating behaviour, the proportions of individuals engaging in binge eating, i.e., devouring large amounts of food, and strict diet and strenuous exercise for weight control were higher than among non-stress-driven eaters. Girls who reported stress-related eating behaviour less commonly had frequent meals with their families, but more commonly shorter sleep duration and regular use of tobacco than their peers who did not seek stress relief from food.

Table 5 shows the fit statistics for the five LCA models tested. Based on the lowest BIC values for boys and girls and the SSABIC value for boys, the three-class solution provided the best fit for data. Although the AIC values progressively decreased with increasing numbers of classes, the LMR values indicated that the four- and five-class models did not improve the model fitting accuracy. However, the LMR statistic of the two-class model, among boys and girls, was equal (girls) or superior (boys) to the three-class solution; the two-class solution was also associated with the highest value of entropy among boys and girls. In the end, we chose the three-class solution on the basis of BIC values and the conceptual meaningfulness of the model. Table 6 presents the clustering of boys and girls according to their health behaviour characteristics into the latent classes I-III that were subsequently named ‘unbalanced weight control’ (Class I), ‘adverse habits’ (Class II) and ‘healthy lifestyle’ (Class III). In the adjusted multinomial logistic regression model (Table 7), both the ‘unbalanced weight control’ and ‘adverse habits’ clusters were associated with an increased risk of overweight among boys and girls. For both genders, the ‘unbalanced weight control’ cluster was also significantly associated with higher odds of obesity.

**Table 5:** Model fit statistics in latent class analysis

**Number of classes in model**	**Statistical indices**				
	**Girls**				
	**AIC**	**BIC**	**SSABIC**	**Entropy**	**LMR *p*-value**
**One class**	40869.835	40937.363	40902.411	N/A	N/A
**Two classes**	39696.616	39737.810	39764.728	0.829	<0.0001
**Three classes**	39192.423	39407.283	39296.071	0.731	<0.0001
**Four classes**	39128.532	39417.059	39267.718	0.701	0.0585
**Five classes**	39094.938	39457.130	39269.659	0.704	0.0152
	**Boys**				
	**AIC**	**BIC**	**SSABIC**	**Entropy**	**LMR *p*-value**
**One class**	32361.412	32427.747	32392.795	N/A	N/A
**Two classes**	31878.669	32017.368	31944.288	0.978	<0.0001
**Three classes**	31756.911	31967.976	31856.766	0.570	0.0007
**Four classes**	31724.121	32007.550	31858.212	0.488	0.0144
**Five classes**	31711.079	32066.873	31879.406	0.536	0.7043

*Abbreviations*: *AIC* Akaike information criteria, *BIC* Bayesian information criteria, *LMR* Lo-Mendell-Rubin likelihood ratio test, *N/A* not applicable, *SSABIC* Sample size-adjusted Bayesian information criteria.

**Table 6:** Clustering of study population into three latent classes based on health behaviours

**Health behaviour**	**Amount or frequency**	**Girls**			**Boys**		
		**Latent class I n = 1256**	**Latent class II n = 1071**	**Latent class III n = 1098**	**Latent class I n = 862**	**Latent class II n = 628**	**Latent class III n = 1583**
**Stress-related eating**	At least to some extent	**732 (58.3%)**	527 (49.2%)	218 (19.9%)	188 (21.8%)	**142 (22.6%)**	136 (8.6%)
**Physical activity**	<2 hours a week	310 (24.7%)	**694 (64.8%)**	384 (35.0%)	126 (14.6%)	**361 (57.5%)**	471 (29.8%)
	2–3 hours a week	407 (32.4%)	252 (23.5%)	**369 (33.6%)**	**229 (26.6%)**	124 (19.7%)	361 (22.8%)
	>3 hours a week	**539 (42.9%)**	125 (11.7%)	345 (31.4%)	**507 (58.8%)**	143 (22.8%)	751 (47.4%)
**Tobacco use**	Never smoked or taken snuff	304 (24.2%)	97 (9.1%)	**735 (66.9%)**	291 (33.8%)	70 (11.1%)	**765 (48.3%)**
	Experimental or occasional use of cigarettes or snuff	**557 (44.3%)**	455 (42.5%)	363 (33.1%)	351 (40.7%)	146 (23.2%)	**704 (44.5%)**
	Smoking or taking snuff regularly	395 (31.4%)	**519 (48.5%)**	0 (0%)	220 (25.5%)	**412 (65.6%)**	114 (7.2%)
**Sleeping**	<8 hours per night	388 (30.9%)	**378 (35.3%)**	72 (6.6%)	167 (19.4%)	**252 (40.1%)**	100 (6.3%)
	8–9 hours per night	735 (58.5%)	549 (51.3%)	**951 (86.6%)**	564 (65.4%)	253 (40.3%)	**1224 (77.3%)**
	≥9 hours per night	133 (10.6%)	**144 (13.4%)**	75 (6.8%)	131 (15.2%)	**123 (19.6%)**	259 (16.4%)
**Binge eating**	Once a month - daily	**167 (13.3%)**	139 (13.0%)	2 (0.2%)	111 (12.9%)	**145 (23.1%)**	64 (4.0%)
**Strict diet to control weight**	Occasionally or often	**792 (63.1%)**	213 (19.9%)	17 (1.5%)	**241 (28.0%)**	35 (5.6%)	27 (1.7%)
**Heavy exercise to control weight**	Occasionally or often	**1256 (100.0%)**	0 (0%)	207 (18.9%)	**862 (100.0%)**	0 (0%)	46 (2.9%)
**Eating meals with one’s family**	A few times a week or less often	574 (45.7%)	**572 (53.4%)**	103 (9.4%)	250 (29.0%)	**300 (47.8%)**	220 (13.9%)

Latent class I = “Unbalanced weight control”; Latent class II = “Adverse habits”; Latent class III = “Healthy lifestyle”.

Boldface was used to indicate the distinguishing features of each latent class.

**Table 7:** Unadjusted and adjusted associations between the three identified health behaviour clusters and obesity measures

**Degree of obesity and latent cluster**	**Girls**				**Boys**			
	**Unadjusted**		**Adjusted**^**a**^		**Unadjusted**		**Adjusted**^**a**^	
	**n**	**OR (95% CI)**	**n**	**OR (95% CI)**	**n**	**OR (95% CI)**	**n**	**OR (95% CI)**
**Normal weight/underweight**	**2939**	Ref.	**2922**	Ref.	**2533**	Ref.	**2513**	Ref.
**Overweight**								
Unbalanced weight control	179	2.24 (1.70, 2.97)	177	2.22 (1.67, 2.94)	181	3.00 (2.35, 3.82)	178	2.98 (2.34, 3.81)
Adverse habits	111	1.54 (1.14, 2.08)	111	1.54 (1.14, 2.09)	73	1.43 (1.06, 1.93)	72	1.42 (1.05, 1.93)
Healthy lifestyle	78	Ref.	78	Ref.	136	Ref.	134	Ref.
	**368**		**366**		**390**		**384**	
**Obesity**								
Unbalanced weight control	48	2.47 (1.44, 4.23)	47	2.49 (1.43, 4.33)	56	3.08 (2.03, 4.65)	55	3.01 (1.98, 4.56)
Adverse habits	31	1.76 (0.99, 3.15)	31	1.79 (0.99, 3.23)	26	1.69 (1.02, 2.79)	24	1.54 (0.92, 2.58)
Healthy lifestyle	19	Ref.	18	Ref.	41	Ref.	41	Ref.
	**98**		**96**		**123**		**120**	

^a^Logistic regression analysis adjusted for maternal basic education level (comprehensive school or upper secondary school) and parental interest in their child’s schooling and hobbies (never/seldom or almost always).

*Abbreviations*: *CI* confidence interval, *OR* odds ratio, *Ref*. reference category.

## Discussion

This study observed an association between stress-related eating behaviour and obesity measures and several unhealthy behaviours including dietary, drinking and weight control habits in a representative, population-based sample of Finnish adolescents. Stress-related eating behaviour was three times more prevalent among adolescent girls than boys. Early life factors, such as birth weight and maternal smoking and drinking during pregnancy were not associated with stress-related eating in adolescence.

The findings are in accordance with previous studies that have found that females, more often than males, report increasing food consumption when stressed or eating certain foods to feel better [30]. When using the same single item belonging to the Ways of Coping Checklist to assess stress-related eating behaviour in adults, the proportion of stress-driven eaters was also higher among women than men [2]. Foods eaten in relation to stress were high-fat or sugary snack foods, which confirmed previous findings [2,6], and people normally avoid these foods for weight control and health reasons [30]. Unhealthy eating has also been associated with avoidant coping style among adolescents [32]. Our results are in line with these previous reports and also bring forth the notion that the association between stress-related eating and obesity exists already in adolescence among both genders. The results lend further support to the notion of eating together with one’s family having a beneficial effect on the health and well-being of the offspring. The effect of family meals seems to be mediated by family cohesion and coping skills [33], thus explaining its inverse association with stress-eating and obesity.

Although early-life programming mechanisms may increase the risk of many diseases, our results do not suggest foetal origins for stress-related eating behaviour, i.e. maternal stress during pregnancy did not seem to increase vulnerability to stress-related eating in adolescent offspring. However, further studies are needed to confirm this. The low prevalence of smoking and alcohol use among pregnant women together with the relatively much higher prevalence of offspring stress-eating behaviour could explain why we did not detect such associations. The other potential indicators of maternal stress, i.e., pre-term birth, low birth weight and smallness for gestational age, were also considerably less prevalent than stress-eating among adolescents.

The co-occurrence of obesogenic habits, i.e., binge eating behaviour, inadequate sleep and consumption of calorie-dense and low-satiety snack foods, could explain the association between stress-eating tendency and greater BMI and waist circumference. Binge eating as defined in this study (eating large quantities of food at least once a month) may indicate features of uncontrolled eating behaviours that seem to be associated with increased BMI and overweight among children and adults [34,35]. Among adolescents and young adults, however, loss of control was found to be a requisite for overeating as a predictor of overweight or obesity [36]. Furthermore, strict diet and strenuous exercise for weight control were associated with stress-related eating behaviour. Drastic weight control methods may be used to compensate for the effect of binge-like or stress-related eating on weight, but it has also been suggested that dieting may contribute to the development of binge eating problems [37]. In fact, extreme weight control behaviours, such as those in the cluster of ‘unbalanced weight control’, have been linked to an increased risk of weight gain and future obesity among adolescents [38].

As well as the ‘unbalanced weight control’ cluster, LCA produced two other distinctive behaviour patterns, ‘adverse habits’ and ‘healthy lifestyle’. The ‘unbalanced weight control’ cluster was characterised by a higher prevalence of eating behaviour abnormalities and extreme weight control practices, whereas low adherence to behaviours known to prevent disease and promote good health was more typical among individuals in another subgroup, accordingly named ‘adverse habits’. This gives rise to the hypothesis that in the treatment of obesity, groups of adolescents with distinct behaviour patterns can be identified using short questionnaires. The content of counselling could be then tailored for these subgroups, e.g. those with adverse habits may need knowledge on health-promoting behaviours, while the other obesity risk groups may benefit from both psychological and dietary therapy.

When stressed, some people increase whereas others decrease their food and drink consumption [3]. A common explanation as to why some individuals eat more after stressful events is restraint eating, i.e., intentional restriction of food intake to maintain or lose weight [39]. Balantekin and Roemmich observed that despite other common stress coping behaviours being freely available, dietary restrained children aged 8–12 ate more when stressed [40]. However, Lowe and Kral suggest that instead of being causally responsible for stress-induced eating, restrained eating represents a proxy risk factor for appetitive hyper-responsiveness and susceptibility to weight gain [41]. As well as cognitive restraint acting as a moderator of stress-induced eating, emotional eating has been linked to increased intakes of energy and fat under stressful conditions [42]. Among children, however, emotional eating did not mediate the stress-diet association [7] and among adolescents, the association between stress and emotional eating was seen only among girls [43]. As regards other psychological factors explaining the stress-eating relationship, cognitive distraction, vulnerability to disinhibition and the type of stressor have been proposed as being influential, in part via interaction with restrained eating [41,42,44,45].

The strength of the study was the large population-based cohort, with high retention rates, which reduces potential selection bias. BMI and waist circumference were based on measured data and were thus free of reporting bias. As regards limitations of the methodology, health behaviours were assessed using non-standardised, self-administered questionnaires. Although not suitable for estimating actual nutrient intake, food frequency questionnaires (FFQs) are appropriate for categorising people according to intake and identifying those at the extremes of intake; moreover, FFQs provide a better approximation of long-term dietary habits compared with short-term food records [46]. FFQs have been widely employed in epidemiological and experimental studies, including studies of children and adolescents [47]. The 24-item FFQ used in this study was specially constructed for the NFBC data collection and was not validated against another dietary assessment method; however, child and adolescent FFQs of medium length (20–60 items) that do not assess portion sizes have been found to obtain higher validity correlates than longer, more detailed ones [48]. In the present study, we focused on frequencies of food intake rather than quantities, due to methodological reasons. Data on energy and nutrient intakes would nevertheless have complemented the results and possibly elucidated associations; however, these were not available for the analyses. While the results demonstrated the feasibility of the single item in the assessment of stress-related eating behaviour and related characteristics, it should be noted that the Ways of Coping questionnaire is not designed for assessing coping styles or traits; it is intended as a process measure to be administered repeatedly, focusing on coping processes in a specific stressful event, and then conducting intraindividual analyses [26]. Given the lack of validity of the measures and the use of cross-sectional data, the results should be interpreted with caution. Regarding reverse causality, it is noteworthy that obesity is associated with adverse social, educational and psychological factors in adolescence, and may be a stressor in itself [49]. Hence, obesity could temporally precede and predispose to stress-induced eating behaviour among adolescents, despite the fact that neither adolescent obesity nor maternal overweight predicted stress-related eating behaviour among young Finnish adults [2].

It is probable that other factors that were not included in the analyses, e.g. psychological traits including restraint and emotional eating behaviour, are associated with stress-related eating behaviour. Although not addressed in the present study, family environment, i.e., home availability of foods, parenting practices and parents’ own eating behaviours, seem to be a crucial determinant of children’s eating styles [50,51]. The development of stress-related eating behaviour may also have a molecular basis: family, twin, adoption and molecular genetic studies suggest that genetic factors have a substantial influence on the liability to develop disordered eating behaviours, including binge eating [52], the heritability of which seems to significantly increase during adolescence [53]. Thus, further studies are needed to describe the etiology of stress-related eating behaviour during childhood and adolescence; the influences of parental models of behaviour and child feeding practices should specifically be investigated. Further studies are also required to find out whether stress-related eating is an indicator of other severe abnormalities in eating behaviour.

In addition to the home environment, many other environments, such as schools, sports clubs and sporting events, can influence adolescents’ food choices; these places often provide easy access to snack-type foods and sugary drinks. Thus, health policies and programmes should be aimed at ensuring the supply of healthy, affordable foods and beverages as well as limiting the availability of energy-dense, low-satiety snacks in these surroundings. For example, modifications in the school food environment have been found to affect adolescents’ food choices both in and outside of school [54].

As the main implication of the results, stress-related eating and several associated unhealthy behaviours indicate an increased risk of adolescent obesity. The results also indicate that the single item used in this study to assess stress-related eating behaviour is an applicable instrument for use in, for example, health examinations at schools, to recognise high-risk subjects who could benefit from intensified counselling on healthy eating and weight management as well as support for health behaviour changes.

## Conclusions

Adolescents reporting stress-related eating consumed sweet and fatty foods and alcohol, binged and used unhealthy weight control methods more often than their peers without stress-driven eating behaviour. Stress-eater girls were especially prone to other unfavourable health behaviours (tobacco use, shorter sleep). Given the tracking of health behaviours from childhood [55], stress-induced eating and drinking in adolescence may lead to using health-compromising coping strategies also in adulthood. Teaching adolescents healthy and effective coping skills to respond to stressors, and addressing co-occurring adverse lifestyles could be a key to preventing unhealthy eating, obesity and obesity-related diseases later in life.

## Abbreviations

AIC: Akaike information criterion; BIC: Bayesian information criterion; BMI: Body mass index; CI: Confidence interval; FFQ: Food frequency questionnaire; IDF: International Diabetes Federation; LCA: Latent class analysis; LMR: Lo-Mendell-Rubin likelihood ratio test; NFBC1986: Northern Finland Birth Cohort 1986; SSABIC: Sample-size-adjusted Bayesian information criterion; WCC: Ways of Coping Checklist.

## Competing interests

The authors declare that they have no competing interests.

## Authors’ contributions

JL, NN, FR, JR and AJ designed the study. MRJ and JL participated in the data collection. JL and JR conducted the statistical analyses. AJ, NN, FR, JR and JL interpreted the results. AJ, NN, MRJ and JL wrote the manuscript. All authors have been involved in the production of the manuscript, and have approved the final version.

## Pre-publication history

The pre-publication history for this paper can be accessed here:

http://www.biomedcentral.com/1471-2458/14/321/prepub
